# Prevalence of Atopic Dermatitis in Chinese Children aged 1–7 ys

**DOI:** 10.1038/srep29751

**Published:** 2016-07-19

**Authors:** Yifeng Guo, Ping Li, Jianping Tang, Xiuping Han, Xiaoyan Zou, Gang Xu, Zigang Xu, Fenglei Wei, Qiang Liu, Min Wang, Fengli Xiao, Wenkai Zong, Chunping Shen, Jianhong Li, Jianzhong Liu, Yongqi Luo, Jing Chang, Nan Sheng, Chun Dong, Duo Zhang, Xing Dai, Jinjie Zhou, Chi Meng, Hongxi Niu, Xuemei Shi, Xinglian Zhang, Juan Xiang, Haitao Xu, Qin Ran, Yi Zhou, Ming Li, Hui Zhang, Ruhong Cheng, Xinghua Gao, Hua Wang, Heng Gu, Lin Ma, Zhirong Yao

**Affiliations:** 1 Department of Dermatology, Xinhua Hospital, Shanghai Jiaotong University School of Medicine, Shanghai, China; 2 Department of Dermatology, Shenzhen Children’s Hospital, Shenzhen, Guangdong, China; 3 Department of Dermatology, Hunan Children’s Hospital, Changsha, Hunan, China; 4 Department of Dermatology, Shengjing Hospital, China Medical University, Shenyang, Liaoning, China; 5 Department of Dermatology, Hubei Maternity and Child Health Hospital, Wuhan, Hubei, China; 6 Department of Community Health and Family Medicine, School of Public Health, Shanghai Jiaotong University, Shanghai, China; 7 Department of Dermatology, Beijing Children’s Hospital, Capital Medical University, Beijing, China; 8 Department of Dermatology, Dalian Children’s Hospital, Dalian, Liaoning, China; 9 Department of Dermatology, Shanxi Children’s Hospital, Taiyuan, Shanxi, China; 10 Department of Dermatology, Chengdu Women’s and Children’s Central Hospital, Chengdu, Sichuan, China; 11 Institute of Dermatology and Department of Dermatology, No.1 Hospital, Anhui Medical University, Hefei, Anhui, China; 12 Institute of Dermatology, Chinese Academy of Medical Sciences, Peking Union Medical College, Nanjing, Jiangsu, China; 13 Pediatric Dermatology, Children’s Hospital of Chongqing Medical University, Chongqing, China; 14 Department of Dermatology, The First Hospital of China Medical University, Shenyang, Liaoning, China

## Abstract

Prevalence of atopic dermatitis (AD) is increasing worldwide. Up to date, there has been no face-to-face nation-wide study in China. We aim to explore the prevalence of clinical diagnosed AD in children aged 1–7 ys in China. Twelve metropolises were chosen from different areas of China. In each region, we selected 4–10 kindergartens and 2–5 vaccination clinics randomly. A complete history-taking and skin examination were performed by dermatologists. The definite diagnosis of AD and the severity were determined by two or three dermatologists. All criteria concerned in UK diagnosis criteria, characteristic presentation of AD and atypical manifestations were recorded in detail. A total of 13998 children from 84 kindergartens and 40 vaccination clinics were included. The prevalence of AD was 12.94% by clinical diagnosis of dermatologists overall, with 74.6% of mild AD. Comparatively, prevalence of AD based on UK diagnostic criteria was 4.76%. This is the first face-to-face nation-wide study in Chinese children aged 1–7 ys, revealing that the prevalence of AD in children is closer to that of wealthier nations.

Atopic dermatitis (AD) is a common, highly pruritic chronic inflammatory skin disorder in children, which is often associated with other atopic diseases such as asthma and allergic rhinitis^1^. Previous study of AD in a hospital-based setting in China revealed that the average onset of AD is 0.86 ± 3.87 years, with 94.6% of them developing AD less than 2 years old^2^. Although not life threatening, intense itching and sleep loss can significantly impact on the quality of life of the individual and his/her family. As a consequence, more than one thousand epidemiological studies related to the prevalence and environmental risk and protective factors of AD were published during the past decades^3^. A systematic review of epidemiological Studies showed the international time trends in the incidence and prevalence of atopic eczema from 1990 to 2010 were increasing in Africa, eastern Asia, western Europe and parts of northern Europe (i.e. the UK)^4^. In Europe, East Germany saw a rise in the number of newly diagnosed AD cases in children up to the age of 6 years from 16.0% in 1991 to 23.4% in 1997, however, the incidence of AD was stable among preschool children in West Germany after the country’s reunification^3^. Prevalence of atopic eczema in 6 to 7-year-old schoolchildren in Spain using ISAAC Phase III in 2010 was 5.92%^5^. In the contrast, another similar study Brazilian schoolchildren show the prevelance ranged from 5.3% to 13.0% among cities^6^. In eastern Asia, a cross-sectional study of 6,453 Korean preschool children illustrated that the AD prevalence was 9.2% determined by dermatological examination 2008^7^. Prevalence of childhood AD is 12–13% in mainland Japan in recent years^8^. In Tokyo, the prevalence of AD was 19.6% and 17.4% in 6- and 7-year old children using ISAAC core written questionnaire in 2005^9^. Up to date, two studies have reported data related to AD prevalence from Chinese population. Both of them were performed by questionnaire investigation using UK diagnostic criteria. One study limited in Shanghai^10^ reported the prevalence of AD in children aged 3–6y in 2012 and another involved 10 cities showed the prevalence in children aged 1–7 ys was 3.07% in 2002^11^. Is the prevalence of AD in China still at a low ebb when compared to surrounding areas? Our primary objective is to determine the actual prevalence of AD in children aged 1–7 ys in China nowadays.

AD has a wide spectrum of dermatological manifestations, including presentation, severity and distribution, which might be atypical or confused in pediatric population. During the past decades, various diagnostic criteria for AD have been proposed^12^. All methodology factors, such as various populations, study methods, can influence the epidemiological result. In the current study, we intended to perform a nation-wide face-to-face study completed by dermatologists to reveal the prevalence of AD in China more definitely.

## Results

### Response Rate and quality of data collection

Of the 15,000 children surveyed, 14478 (96.52%) finished questionnaire and were examined by dermatologists. 480 individuals were excluded by data analysis due to overage or incomplete information. 335 individuals were less than 1-year-old and 22 individuals were over 7-year-old. Another 123 cases didn’t give full information. Finally, a total of 13998 preschool children were included in this study.

### Population characteristics

The epidemiological study was performed in 84 kindergartens and 40 vaccination clinics in 12 metropolises throughout China. The characteristics of 13998 children are listed in Table 1. There were no significant differences in these characteristics between the AD and non-AD groups.

### Prevalence of AD

**Figure 1:**
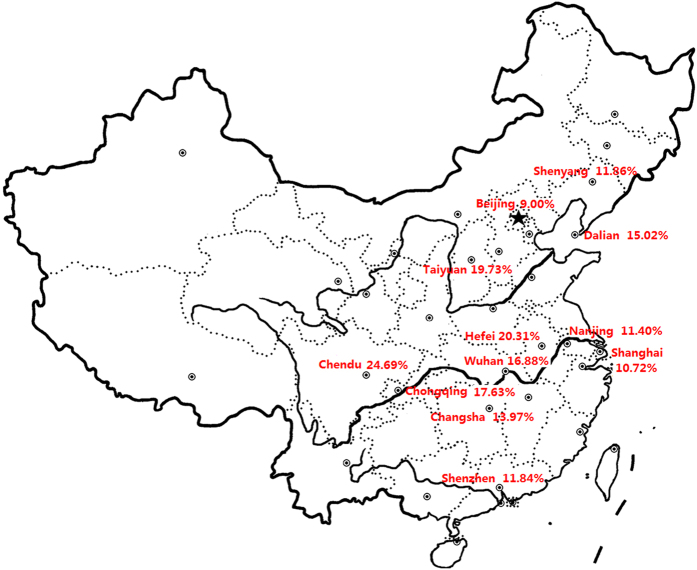
AD prevalence of children aged 1–7 ys in China (The figure is created using mapinfo made in China http://www.downxia.com/downinfo/50308.html and Adobe PhotoShop CS6 softwares http://rj.baidu.com/soft/detail/23675.html?ald).

The point prevalence of AD based on clinical diagnosis by dermatologists was 12.94% overall (1811 of 13998), ranging from 9.00% to 24.69% between metropolises for preschool children in China (Fig. 1 and Table 2). The proportion of mild, moderate and severe AD is 74.60%, 23.96%, and 1.44% respectively (Table 3). Of all 1531 mild AD, 780 cases presented with xerosis. The highest district prevalence values were reported in inland area of China, such as Chengdu (24.69%), Hefei (20.31%) and Taiyuan (19.73%). The lowest prevalence values were in the north and east part of the country (Fig. 1 and Table 2). The prevalence is decreasing along with the growth of age on the whole (Table 4). The Cohen’s kappa coefficient for total subjects was 0.69 and it varies between 0.5 and 0.8 in 12 metropolises (Table 2). Pearson correlation test indicated that no correlation was detected between the kappa coefficients and the AD prevalence by the clinical diagnosis(r = 0.03, p = 0.925). Comparatively, the prevalence of AD based on UK diagnostic criteria for children aged 1–7 yrs was 4.76% (667/13998). The sensitivity, specificity, positive predictive value and negative predictive value were 36.27%, 99.91%, 98.5%, and 91.34% for UK diagnostic criteria. In addition, 2 psoriasis vulgaris and 106 contact dermatitis were diagnosed. No scabies, hereditary metabolic disease or lymphoma were found.

### Risk factors for AD

Residence in rural and an older age are both protective factors for AD. Exposed to passive smoking (especially exposed to also during pregnancy), premature birth, and choosy in food are risk factors for AD. Pet ownership, delivery pattern and feeding pattern in the first 6 months after birth are not shown to be associated with AD prevalence in logistic regression analysis (Table 5).

## Discussion

AD is a global public health concern considering its increasing prevalence and impact on life quality of patient and family. The ISAAC (International Study of Asthma and Allergies in Childhood) revealed that AD affects children across the globe, although the disease prevalence varies substantially between countries^13^. The prevalence of AD is also increasing, especially in developing countries^13^. The current study is the first face-to-face nation-wide study conducted by dermatologists in Chinese children aged 1–7 ys.

AD prevalence at 12.94% in Chinese children aged 1–7 ys is very close to that of Japan and Korea^14^,^15^, but it is much higher than two previous Chinese studies of AD prevalence, both of which used UK diagnostic criteria. The first study was conducted in 2002 by distributing 49241 questionnaires to 10 provincial capitals, including 6 cities in our study, and showed that the prevalence of AD in children aged 1–7 ys was 3.07%^11^. The second study was conducted in 8 communities of Shanghai in 2010 by questionnaire, and showed that prevalence of AD was 8.3% in children aged 3 to 6 in Shanghai^10^. Except for sample variance and fluctuation of prevalence with time, another two factors, the way of data collection and selection of different diagnostic standards, also lead to discrepancy of our study and previous ones. There have been numerous epidemiological studies of AD using questionnaires, but few studies have been performed by dermatologists’ physical examinations owing to much time and cost involved^14^. Secondly, we used clinical diagnosis by experience dermatologists, which is in general considered as gold standard^12^, as the standard but not any criteria in the epidemiological survey. In order to guarantee uniformity of subjective diagnosis, a panel of experienced dermatologists enrolled in skin examination. In addition, every single diagnosis of AD was made by 2 or 3 dermatologists to avoid fallibility and guarantee accuracy of data. The total Cohen’s kappa coefficient at 0.69 showed a relatively good consistency. A relatively higher proportion of mild AD in Chongqing patients can partly explain the relatively low kappa coefficient at 0.51. No correlation between the kappa coefficient and the AD prevalence based on clinical diagnosis indicated low possibility of over-diagnosis by the third dermatologist. Two patients with psoriasis vulgaris and 106 patients with contact dermatitis, whereas no scabies, hereditary metabolic disease or lymphoma was found. All subjects diagnosed with contact dermatitis had the skin lesion only localized to perioral or perianal region for no more than 3 months. All authors regard the dermatologists’ diagnosis as correct and trustable in the present study. When UK diagnostic criteria used, the prevalence of AD in children aged 1–7 ys according to data collected by dermatologists in our study seems quite close to that from questionnaires 10 years ago(4.76% compared to 3.07%). However, if clinical diagnosis of dermatologists adopted, the prevalence of AD in the current study is much higher than previous data using UK diagnostic criteria (12.94% compared to 3.07%).

Little is known about the distribution of AD severity in Chinese population and abroad. Current study showed that about 74.60% affecters presented with mild lesions. 23.96% of AD is moderate and 1.44% of AD is severe. In epidemiology of 0–17 childhood atopic dermatitis in US, 67% of children had mild, 26% had moderate, and 7% had severe disease throughout the country^16^. Another study of 290 children aged 1–5 year preschool children show that 82% had mild, 12% moderate, and 6% severe disease of AD in 2000. Also a study using clinician assessment of severity found 84% mild, 14% moderate and 2% severe in 1760 children aged 1–5 year in 1998^17^. The distribution of AD severity in China seems similar to that in abroad.

Prevalence of AD ranges from 9.00% to 24.69% among cities. A general geographic trend of higher disease prevalence in inland area of China was found. It is noted that inland cities accelerate industrialization in the past year. Shenzhen and Hong Kong are two cities next to each other geographically. Prevalence of AD in Shenzhen aged 1–7 ys is currently 11.84%, compared with 14% in children aged 6–12.5 ys in Hong Kong in 2000^18^. In another aspect, the prevalence of AD is highest among 1–2 year old children and descends with age in general, which is in accordance with natural advance of AD. Prevalence of AD fluctuates with age, indicating that multiple factors play a role in AD development.

A series factors including age, residence status, exposed to passive smoking, premature birth, breast-feeding, pet ownership, delivery pattern and choosy in food were previously considered to be associated with AD prevalence and evaluated in multiple studies^19^,^20^,^21^,^22^,^23^,^24^,^25^,^26^,^27^,^28^,^29^. AD prevalence is generally considered declined with age and to be lowered in rural areas, which is consistent with current study. For breast-feeding, there is strong evidence to support that breast-feeding during the first 4 months of life causes a reduction in incidence and severity of atopic disease in patients at high risk, however, the risk reduction from breast-feeding only applies to children at high risk^24^. Besides, there were conflicts on the impact of passive smoking on AD (both as risk and protective factors^19^,^20^,^21^,^22^), premature birth (decreased risk for severe atopic dermatitis^23^), pet ownership (protective influence being under investigation^26^,^28^), delivery pattern^27^ and choosy in food^25^. In our study, passive smoking, premature birth, and choosy in food are risk factors for AD. Pet ownership, delivery pattern and feeding pattern in the first 6 months of life are not influence factors for AD development, which somewhat enriches the information on controversial risk factors for Chinese pediatric AD, and indicated that more exact studies are required.

One of the earliest and most recognized sets of diagnostic criteria is the 1980 Hanifin and Rajka criteria^30^. While widely used in clinical trials, it is not convenient for use in clinical practice because of comprehensive criteria. Several international groups have proposed modifications to address limitations (eg, Kang and Tian criteria, International Study of Asthma and Allergies in Childhood [ISAAC] criteria). The United Kingdom (UK) Working Party, in particular, systematically distilled the Hanifin and Rajka criteria down to a core set that is suitable for epidemiologic/population-based studies and that can be used by non-dermatologists^31^. It consists of 1 mandatory and 5 major criteria and does not require any laboratory testing. The UK Working Party diagnostic schemes have been validated in studies and tested in different populations^32^,^33^,^34^,^35^,^36^. It is noted that there is a huge discrepancy in the AD prevalence between the dermatologists’ diagnosis (12.94%) and the UK diagnostic criteria (4.76%). Using clinical diagnosis as gold standard, the sensitivity, specificity, positive predictive value and negative predictive value were 36.27%, 99.91%, 98.5%, and 91.34% for UK diagnostic criteria. Compared to previous studies^37^,^38^, a relatively low sensitivity was also shown. The cause for the low sensitivity can be explained as following. In the current study, it is noted that AD patients at an early age with mild phenotype are easy to be omitted when diagnosed by UK diagnosis criteria. For example, 63.17% AD patients diagnosed by dermatologists were not approved when UK diagnosis criteria worked. Among them, 84.97% were mild AD with the objective SCORAD value less than 14.8 while other 15.03% of them show that their objective SCORAD value was between 15 and 53.5. Among missed diagnosed patients, 43.33% of them didn’t report persistent pruritis over 12 months, which is a mandatory criterion in UK diagnosis criteria. 51.29% of them denied a generally dry skin in the last year. Both were not easy for carers to judge and reply accurately in practice, especially for children less than 1 year old. 98.10% denied personal asthma or hay fever, which is rarely seen or diagnosed in children less than 3 year old. 89.80% of them did not show rash in the skin creases. 6.94% were normal before 2 years old. Therefore, it was also speculated that all above made missed diagnosis when UK diagnosis criteria adopted.

In conclusion, this is the first face-to-face nation-wide study in Chinese preschool children conducted by dermatologists to evaluate the prevalence of AD, revealing that the prevalence of AD in children aged 1–7 ys is close to that of wealthier nations.

## Methods

### Study population

This epidemiologic study used a population-based stratified random sample of 15000 individuals. The target population consisted of preschool children with age range 1–7 years in kindergartens (age 4–7 years) and vaccination clinics (age 1–3 years), considering that the rate of kindergarten attendance is over 90% according to data from local Bureau of education while vaccination rate is over 95% according to data from local center for disease control and prevention among 1–7 year old children in metropolis. For nation-wide study, 12 metropolises were chosen from different areas of China: Shenyang, Beijing, Dalian, Taiyuan, Nanjing, Hefei, Shanghai, Wuhan, Chengdu, Chongqing, Changsha and Shenzhen, respectively, throughout China (Fig.1). In each region, we randomly selected 4–10 kindergartens and 2–5 vaccination clinics. The precise numbers of kindergartens, vaccination clinics and samples enrolled in epidemiological survey were decided statistically according to demographic data of children among 1–7 years in each city when prevalence of AD was estimated at about 10%.

### Diagnostic criteria of AD

All dermatologists involved in study have rich clinical experience in AD and have been trained to unify their recognition of AD in advance. In this study, clinical diagnosis made by experienced dermatologists was considered as gold standard^12^. The clinical diagnosis is essentially following the definition of AD, a chronic, relapsing, highly pruritic, inflammatory skin disease that frequently predates the development of allergic rhinitis or asthma^39^; moreover, other allergic, pruritic, or erythematosquamous dermatosis, like contact dermatitis, psoriasis, scabies, or hereditary metabolic disease and lymphoma have been carefully excluded by history inquiring (Questions included “when were the skin disorder developed and how long it has been existing” “when was the disease most severe” “what do you consider as the predisposing factors” “Has family members affected by same disease?”) and physical examination (all positive signs of skin were recorded). No laboratorial test was performed for dermatologists to make the diagnosis.

### Data collection

The study and related questionnaire were approved by the Ethical Committee of Xinhua Hospital affiliated to Shanghai Jiaotong University School of Medicine. Permission was also obtained from Center for Disease Control and Prevention (CDC) and school principals. Written Informed consent was provided by all children’s parents. Data collection was carried out in accordance with the principles of the Declaration of Helsinki and approved guidelines. From December 2013 to February 2014, dermatologists visited schools or vaccination clinics and independently evaluated whether or not the child had AD and the severity. Each subject was inspected by 2 dermatologists independently at first. Each dermatologist was given an equal and independent authority to make their own diagnosis after a 10–15 minutes’ dermatological physical examination. One dermatologist did not know another dermatologist’s diagnosis, whether each subject met the UK Criteria, or other physician’s idea in advance. If inconsistency exists between 2 dermatologists, the idea of another senior dermatologist will be adopted after examining the patient again. Afterwards, two dermatologists evaluated the severity of each AD patients according to “SCORing Atopic Dermatitis” (SCORAD), a clinical tool for assessing the severity of atopic dermatitis objectively, independently and the average score would be the final result. The severity of AD is evaluated and classified as mild (<15), moderate (15–40) or severe (>40) according to the objective components of the SCORAD index (clinical signs and disease extent, total score 83). All diagnostic criteria mentioned in UK diagnostic criteria as well as personal and family history of atopic diseases including infantile eczema were investigated and recorded in detail for determination of AD incidence based on UK diagnosis criteria(Parents or guardians filled the relevant forms). All characteristic presentation of AD as well as atypical clinical manifestations were also inspected and recorded in detail for all subjects. General information, personal and family history of skin diseases including allergic/atopic disorders and the subjective symptom of pruritus were collected from parents or other family members by dermatologists on the examination day in vaccination clinics or one week ahead for children in kindergartens.

### Risk factor

Possible risk factors^19^,^20^,^21^,^22^,^23^,^24^,^25^,^26^,^27^,^28^,^29^ for AD included age, residence status (urban or rural district), passive smoking (not exposed to, exposed to but not during pregnancy, exposed also including pregnancy time), pet ownership (not exposed to, exposed to but not during pregnancy, exposed also including pregnancy time), delivery pattern (vaginal delivery or caesarean), premature birth or not, feeding pattern during the first 6 month after birth (exclusive breast-feeding, mixed feeding, powdered milk), and choosy or not in food. So we evaluated the factors in the epidemiological study.

### Statistical analysis

The prevalence of AD in various age groups and cities, as well as the severity of AD was calculated separately. The consistency between the diagnosis of two dermatologists was examined by Cohen’s kappa coefficient. Pearson correlation test was used to evaluate the correlation of Cohen’s kappa coefficients and AD prevalence of various cities diagnosed by clinical dermatologists. Logistic regression using the forward likelihood ratio was performed to explore risk factors of AD by data from dermatological examination and questionnaire survey. A *p*-value at less than 0.05 is considered statistically significant. Compared to dermatologist diagnosis, the sensitivity, specificity, positive predictive value and negative predictive value were calculated for UK diagnostic criteria.

## Additional Information

**How to cite this article**: Guo, Y. *et al*. Prevalence of Atopic Dermatitis in Chinese Children aged 1–7 ys. *Sci. Rep.* **6**, 29751; doi: 10.1038/srep29751 (2016).

## Figures and Tables

**Table 1:** Demographic characteristics of study population.

Characteristics	Study population (*n* = 13998)	AD group (*n* = 1811)	Non-AD group (N = 12187)
Age, mean ± SD (ys)	3.56 ± 3.10	3.15 ± 1.70	3.62 ± 3.26
Range (ys)	1–6.83	1–6.50	1–6.83
Gender (%)			
Male	6461 (46.16%)	869 (47.98%)	5854 (48.03%)
Female	7537 (53.84%)	942 (52.02%)	6333 (51.97%)

SD, standard deviation; ys, years.

**Table 2:** AD prevalence of children aged 1–7 ys by metropolis.

Metropolis	Children surveyed	Diagnosis of AD	Prevalence (%)	Cohen’s kappa coefficient
Chengdu	482	119	24.69	0.69
Hefei	517	105	20.31	0.73
Taiyuan	603	119	19.73	0.70
Chongqing	556	98	17.63	0.51
Wuhan	764	129	16.88	0.72
Dalian	679	102	15.02	0.80
Changsha	1260	176	13.97	0.66
Shenyang	1037	123	11.86	0.59
Shenzhen	1504	178	11.84	0.75
Nanjing	1026	117	11.40	0.65
Shanghai	2547	273	10.72	0.71
Beijing	3023	272	9.00	0.68
**Total**	**13998**	**1811**	**12.94**	**0.69**

**Table 3:** Severity of atopic dermatitis.

AD severity (Objective SCORAD)	NO.	%
Mild (<15)	1351	74.60
Moderate (15–40)	434	23.97
Severe (>40)	26	1.44

**Table 4:** AD prevalence stratified by age subgroup.

Metropolis	Prevalence stratified by age subgroup											
	1–2 ys		2–3 ys		3–4 ys		4–5 ys		5–6 ys		6–7 ys	
	N	AD (%)	N	AD (%)	N	AD (%)	N	AD (%)	N	AD (%)	N	AD (%)
Chengdu	62	24 (38.71)	28	10 (35.71)	77	28 (36.36)	84	19 (22.62)	117	13 (11.10)	114	25 (21.93)
Hefei	84	24 (28.57)	51	17 (33.33)	73	10 (13.70)	129	25 (19.38)	140	25 (17.86)	40	4 (10.00)
Taiyuan	108	31 (28.70)	81	16 (19.75)	113	25 (22.12)	129	20 (15.50)	125	18 (14.40)	47	9 (19.15)
Chongqing	130	39 (30.00)	63	14 (22.22)	113	16 (14.16)	97	13 (13.40)	108	10 (9.26)	45	6 (13.33)
Wuhan	98	29 (29.59)	56	9 (16.07)	180	28 (15.56)	190	32 (16.84)	167	25 (14.97)	73	6 (8.22)
Dalian	152	31 (20.39)	84	13 (15.48)	171	14 (8.19)	140	23 (16.43)	69	13 (18.84)	63	8 (12.70)
Changsha	240	18 (7.50)	198	20 (10.10)	218	34 (15.60)	236	47 (19.92)	270	44 (16.30)	116	17 (14.66)
Shenyang	198	27 (13.64)	115	12 (10.43)	214	28 (13.08)	257	29 (11.28)	149	16 (10.74)	106	11 (10.38)
Shenzhen	282	61 (21.63)	168	17 (10.12)	225	22 (9.78)	334	32 (9.58)	382	37 (9.69)	98	13 (13.27)
Nanjing	117	37 (31.62)	91	16 (17.58)	207	19 (9.18)	226	11 (4.87)	252	25 (9.92)	133	9 (6.77)
Shanghai	449	82 (18.26)	271	26 (9.59)	352	32 (9.09)	562	56 (9.96)	579	52 (8.98)	334	25 (7.49)
Beijing	593	98 (16.53)	295	37 (12.54)	519	43 (8.29)	720	30 (4.17)	559	33 (5.90)	337	31 (9.20)
**Total**	**2513**	**501 (19.94)**	**1501**	**207 (13.79)**	**2462**	**299 (12.14)**	**3104**	**337 (10.86)**	**2917**	**311 (10.66)**	**1501**	**156 (10.39)**

**Table 5:** Logistic regression of factors associated with AD.

Risk factor	Classification	AD prevalence by the presence of the risk factor	P value	OR (95%CI)
Increasing of age	—	—	<0.001**	0.829 (0.802–0.857)
Residence status (rural)	urban	8738 (62.42%)	<0.001**	0.086 (0.071–0.105)
	rural	5260 (37.58%)		
Exposed to passive smoking	not exposed to	7384 (52.75%)	0.023*	1.076 (1.010–1.145)
	exposed to but not during pregnancy,	3485 (24.90%)		
	exposed also including pregnancy time	3129 (22.35%)		
Pet ownership	not exposed to	12692 (90.67%)	0.75	0.983 (0.887–1.091)
	exposed to but not during pregnancy,	570 (4.07%)		
	exposed also including pregnancy time	736 (5.26%)		
Delivery pattern	vaginal delivery	6857 (48.99%)	0.21	1.077 (0.971–1.194)
	caesarean section	7141 (51.01%)		
Premature birth	No	12836 (91.70%)	0.001**	1.334 (1.124–1.584)
	Yes	1162 (8.30%)		
Giving up exclusive breast-feeding during first 6 months of life	exclusive breast-feeding	8491 (60.66%)	0.083	0.941 (0.881–1.004)
	mixed feeding	2613 (18.67%)		
	powdered milk	2894 (20.67%)		
Choosy in food	No	10293 (73.53%)	<0.001**	1.272 (1.135–1.425)
	Yes	3705 (26.47%)		

^*^P < 0.05, **P < 0.01. All the risk factors were included in a single formula.
